# Endodontic and prosthetic treatment of teeth with periapical lesions in a 16-year-old-girl

**DOI:** 10.1590/S1678-77572010000200016

**Published:** 2010

**Authors:** Buket AYNA, Emrah AYNA, Sema ÇELENK

**Affiliations:** 1 DDS, PhD, Assistant Professor, Department of Pedodontics, Faculty of Dentistry, Dicle University, Diyarbakir, Turkey.; 2 DDS, PhD, Associate Professor, Department of Prosthodontics, Faculty of Dentistry, Dicle University, Diyarbakir, Turkey.; 3 DDS, PhD, Associate Professor, Department of Pedodontics, Faculty of Dentistry, Dicle University, Diyarbakir, Turkey.

**Keywords:** Periapical lesion, Calcium hydroxide, Ribbond R. Polyethylene fiber

## Abstract

This paper reports the nonsurgical endodontic therapy using calcium hydroxide intracanal dressing and prosthetic treatment of 9 teeth with periapical lesions in a 16-year-old female patient. The periodontal treatment plan included oral hygiene instructions, mechanical debridement and gingivectomy in the maxillary incisors to improve gingival contouring. Root canal treatment was indicated for teeth 11-13, 21, 22, 42-45. After successive changes of a calcium hydroxide intracanal dressing during 6 weeks, the size of the periapical radiolucencies decreased and lesion remission occurred after root canal obturation. The endodontically treated teeth received a bondable polyethylene reinforcement fiber (Ribbond) in the prepared canal space and crown buildup was done with composite resin. Prosthetic rehabilitation was planned with single-unit metal-ceramic crowns and fixed partial dentures. Clinical and radiographic evaluation after 6 months showed successful results. The outcomes of this case showed that chronic periapical lesions can respond favorably to nonsurgical endodontic treatment in adolescent patients and that, with proper indication, polyethylene fibers can provide an effective conservative and esthetic option for reinforcing endodontically treated teeth undergoing prosthetic rehabilitation.

## INTRODUCTION

A complication of deep caries is pulp necrosis. After necrosis develops, bacteria invading the root canal play a decisive role in the development of a periapical lesion^2,27^. Current approaches in the treatment of teeth with periapical lesions include initial nonsurgical root canal treatment. When this treatment is unsuccessful in resolving the periradicular pathology, additional treatment options should be considered^02,24^. In some situations, nonsurgical treatment may be ineffective or difficult, and these cases may be treated surgically^2,4^. Younger patients have better treatment outcomes than older patients^2,12^.

Some authors believe that if the endodontic infection is eliminated, the immune system can promote lesion repair^6,17,23^. Calcium hydroxide is an effective intracanal antibacterial dressing, owing mainly to its high pH and its ability to destroy bacterial cell walls and protein structures^2,5^. Treatment with calcium hydroxide as an interim dressing in the presence of large, chronic periapical lesions can create a more favorable environment to healing and can encourage bone repair^2,9^.

Generally, the successful treatment of teeth with pulpal disease depends not only on good endodontic therapy, but also on good reconstruction of the teeth after the endodontic therapy is completed^26^. It has been suggested that endodontically treated teeth are more brittle and fracture more easily than vital teeth^1,16^. The placement of an intraradicular post in the root canal after endodontic treatment has been recommended to strengthen or reinforce the tooth. However, several studies have pointed out that posts do not strengthen teeth and that post space preparation and post cementation can weaken the root, possibly leading to its fracture^10,20,25^. Therefore, the evaluation of whether a post is needed is based on how much natural tooth substance remains to retain a core buildup and support the final restoration after caries removal and endodontic treatment have been completed. The ideal post and core material should have physical properties that are similar to those of dentin, including its modulus of elasticity, compressive strength, and coefficient of thermal expansion^26^.

Adhesive restorations allow clinicians to create minimally invasive preparations, thereby preserving sound tooth structure^15^. Ultra-highmolecular-weight polyethylene (UHMWPE) fiber reinforcement systems are gaining popularity and have various clinical applications. Being bondable reinforcement fibers, they can be used to build up endodontic posts and cores, as they adapt to the root canal walls without requiring additional enlargement of the root canal after endodontic treatment. These woven fibers have a modulus of elasticity similar to that of dentin and are thought to create a monoblock dentin-post-core system, which produces a better distribution of forces along the root^8^.

Several prosthetic treatment modalities are possible after completing the root canal therapy. Most of them involve either crowns or direct restorations such as resin composites, amalgam, or cements^18^. In endodontically treated teeth with excessive structural loss, this condition must be considered when planning a complete crown. Tooth reduction for placement of an artificial crown is considerable, particularly when teeth are prepared for metal-ceramic crowns^3^. Due to esthetic reasons, this is usually the method of choice for crown coverage of premolars and anterior teeth^29^.

This paper reports the nonsurgical endodontic therapy and prosthetic treatment of multiple teeth with periapical lesions in a young patient.

## CASE REPORT

### Patient and Treatment Plan

A 16-year-old female patient, who lives in a village 180 Km from Diyarbakir in Turkey, was referred to the Department of Pediatric Dentistry of the Faculty of Dentistry, Dicle University, for treatment of severe pain in the region of tooth 43. The girl was the fifth child in a very poor family of nine children with no education. Written informed consent was obtained from the patient and her parents for treatment and publication of the case.

**Figure 1:**
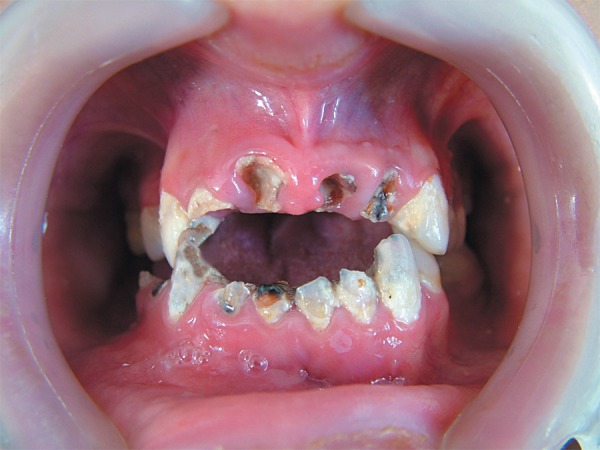
Preoperative intraoral view of teeth

**Figure 2:**
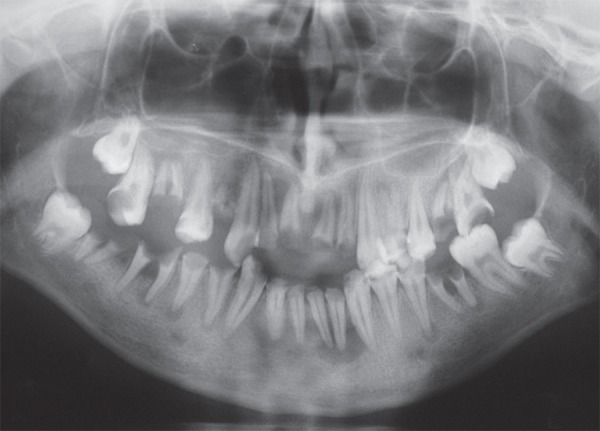
Preoperative panoramic radiograph showing teeth with periapical lesions

Clinical and radiographic examinations showed that she had deep caries, periapical radiolucent areas, and periodontal pockets (Figures 1 and 2). Teeth 14, 16, 26, 31, 32, 36, 46, and 47 had to be extracted because of the severity of the bone lesion and excessive structural loss. Moreover, root canal treatment was indicated for teeth 11-13, 17, 21, 22, 24, 27, 35, and 41-45. However, the girl’s parents stated that travelling to our clinic was difficult for them and asked to have the necessary teeth extracted in a hospital closer to their village. So, analgesics and antibiotics were prescribed for the severe pain in the region of tooth 43 and the patient was discharged. When she returned, it was realized that a dental technician had unfortunately extracted teeth 17, 24, 27, 35, and 48 by mistake. Therefore, the treatment plan had to be changed and we persuaded the parents to have their daughter undergo treatment at our school. Following a thorough oral and periodontal examination, a treatment plan was developed that included oral hygiene instructions, mechanical periodontal debridement, and periodontal reevaluation. Gingival contouring was improved with gingivectomy in the maxillary incisors. Teeth 15, 25, 33, and 34 were restored with composite resin (Clearfil AP-X; Kuraray Company Ltd., Tokyo, Japan) and tooth 37 was restored with amalgam (Spheredon-M, CE, Yehuda, Israel).

**Figure 3:**
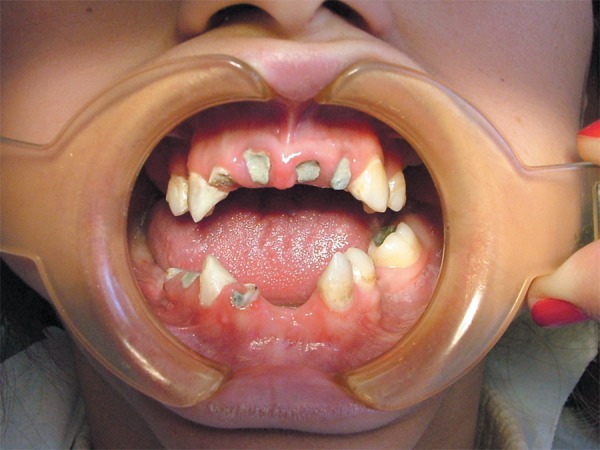
Postoperative intraoral view of teeth

**Figure 4:**
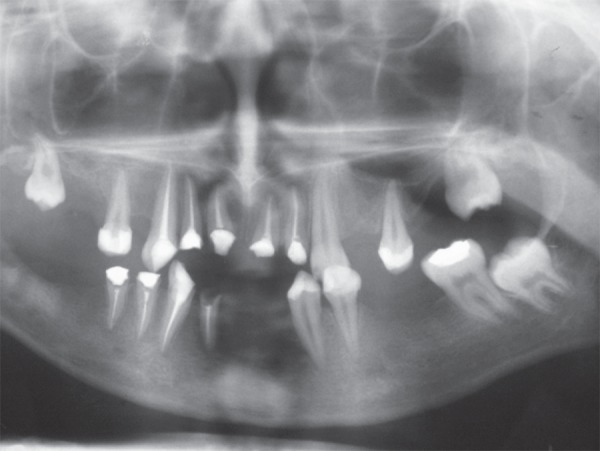
Panoramic radiograph taken 6 months after the endodontic treatment. Note a remarkable decrease of the radiolucencies

Root canal treatment was planned for teeth 11-13, 21, 22, and 41-45. However, tooth 41 had to be extracted because no healing was observed after treatment (Figure 3). At the first appointment, following access cavity preparation, the remaining vital pulp tissue was extirpated and mucopurulent fluid was drained from the root canals of teeth 41-43. The working lengths were estimated as being 1 mm short of the radiographic apices. The root canals were prepared with Kfiles, irrigated with copious amounts of 2.25% sodium hypochlorite, and dried with paper points. After complete instrumentation, a calcium hydroxide paste (Vision, Germany) with a powderto-glycerin ratio of 8:1 was placed in the canals with lentulo filler. The access cavities were sealed with zinc oxide-eugenol cement. The intracanal dressing was changed weekly during 6 weeks. After this period, the teeth were symptom free and the root canals were dry. A continuous decrease in the size of the periapical radiolucencies was observed. Then, the root canals were obturated with gutta-percha (Gapadent, Tianjin, China) and a resin-based root canal sealer (Diaket; 3M ESPE, Seefeld, Germany) using a lateral condensation technique. The patient returned for clinical follow-up after 6 months, and radiographic evidence of lesion remission was observed (Figure 4).

**Figure 5:**
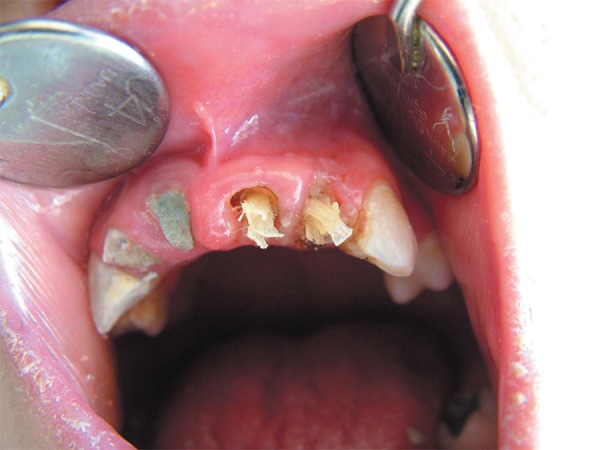
Clinical view of the bondable polyethylene reinforcement fiber (Ribbond) in position

In preparation for crown buildup, 3 to 4 mm of gutta-percha were removed from the canal to expose the dentin. This procedure has been shown to increase microretention when using enamel dentin adhesive systems. The necessary width of the bondable polyethylene reinforcement fiber (Ribbond; Ribbond, Inc., Seattle, WA, USA) was determined to be 2 mm. The prepared dowel space was measured with a periodontal probe, and the measurement was tripled to determine the length of fiber required. Two pieces of fiber were then cut with Ribbond shears (Ribbond, Inc.), coated with a dual-cured resin composite (Liner Bond II V; Kuraray Company Ltd.), and placed in a lightproof container. The internal surfaces of the root canal and pulp chamber were treated with primer (Liner Bond II V, primer A and B mixture; Kuraray Company Ltd.) for 30 s and dried under a gentle stream of air for 15 s. A dual-cured dentin bonding agent (Liner Bond II V, bond A and B mixture; Kuraray Company Ltd.) was applied to the internal surfaces of the canal and pulp chamber and was thinned with a brush. Then, a highly filled, dualcured hybrid resin cement (Panavia-F; Kuraray Company Ltd.) was injected into the canal space. One piece of the reinforcement fiber, which had been coated with bonding agent, was folded and packed into the canal space as tightly as possible, using an endodontic plugger. The second piece was then packed into the canal space perpendicular to the first. The excess resin was removed, and the core buildup was completed using a light-cured hybrid resin composite (Clearfil AP-X; Kuraray Company Ltd.) (Figure 5).

Complete rehabilitation of the dentition was planned. It was decided that teeth 11, 12, 21 and 22 would receive single-unit metal-ceramic crowns; teeth 33, 42 and 43 would support a 6unit metal-ceramic fixed partial denture; teeth 44 and 45 and the pontic tooth 46 form a 3-unit metal-ceramic cantilever fixed partial denture; and teeth 34 and 37 would support a 4 four-unit metalceramic fixed partial denture. Prosthetic treatment of the edentulous areas in the posterior maxilla was delayed until teeth 18 and 28 erupted in the oral cavity because they were posterior abutment teeth.

**Figure 6:**
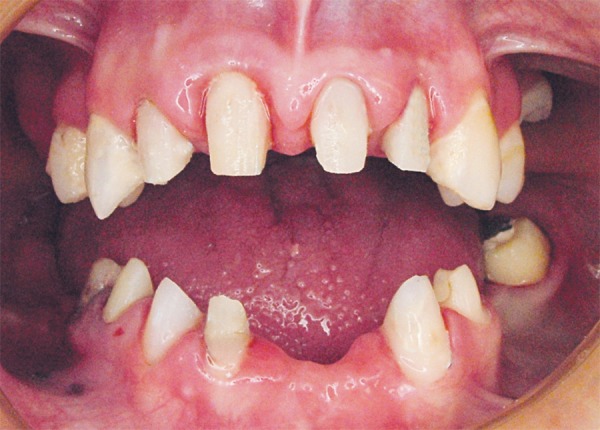
Clinical view of crown preparation

**Figure 7:**
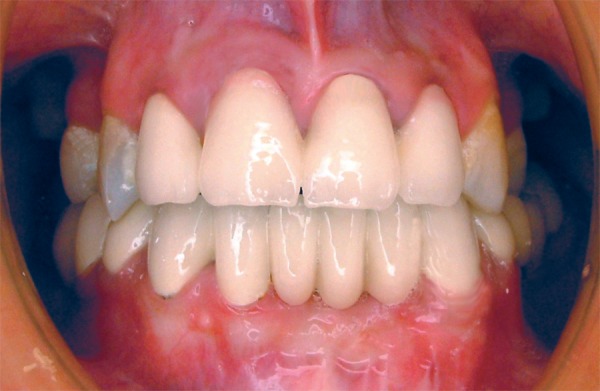
Clinical view of the definitive restoration

For the metal-ceramic crown group, the crowns were prepared 1 week after crown buildup (Figure 6), in a conventional manner using a diamond bur (ISO n 836; Komet, Rock Hill, SC, USA) with a convergence of approximately 2.0 degrees with the aim of obtaining a 6-degree convergence between walls^15^. All teeth were prepared with a long chamfer, and all margins were placed at the gingival level. Occlusal reduction and the crown margins were prepared using a diamond bur (ISO n 836; Komet), and a minimum of 1 mm of coronal dentine was left above the chamfer. Impressions were made with a polyether impression material (Permadyne; 3M ESPE) using a custom tray. After the impression procedure, a temporary resin crown was immediately adapted and cemented on the prepared tooth with zinc oxide eugenolbased temporary cement (Temp Bond; Kerr Corp., Orange, CA, USA). The casting try-in was performed 1 week after the impression was made. After the casting try-in, the porcelain was applied. The crowns were cemented 1 week after the casting try-in, using zinc oxide eugenol-based temporary cement (Temp Bond; Kerr). Two weeks after the temporary cementation, the crown was removed and definitive cementation was performed with zinc phosphate cement (SS White cement; SS White, Glaucester, UK) (Figure 7).

The patient was recalled after 6 months for clinical and radiographic evaluation and good treatment outcomes were observed.

## DISCUSSION

The treatment of teeth with excessive structural loss and pulp necrosis is considered difficult. However, as shown in this case, the healing of periapical lesions is rapid after multiple dressings with calcium hydroxide. Sjögren, et al.^22^ (1991) found that the use of calcium hydroxide dressings for 1 week efficiently eliminated bacteria in infected root canals. Calcium hydroxide is readily available, simple to prepare, resorbable, and more soluble in glycerin than in water. To produce more available hydroxyl ions for the antibacterial effect, coating the root canals with a glycerin/calcium hydroxide paste is superior to water^2,4^. According to previous histopathological results, better apical and periapical tissue repair was observed in groups treated with calcium hydroxide intracanal dressings compared to those treated with immediate obturation without any dressing^13^. Recent studies have shown that teeth that receive intracanal medication present a less intense inflammatory periapical reaction, which confirms the importance of dressing in the endodontic treatment of teeth with pulp necrosis and periapical lesions^9,13,22^.

In the present case, healing of the periapical lesions was observed very soon after applying the calcium hydroxide dressings. Radiographic signs such as changes in the density of the lesions, trabecular reformation, and lamina dura reformation confirmed healing, demonstrating that the teeth were symptom free and that the soft tissues were healthy^24^. The rapid healing response observed in the present case might be attributed to the high healing potential of young patients. In previous reports, treatment with calcium hydroxide resulted in high rates of periapical healing, and some lesions, especially in young patients, were reduced or had disappeared completely 1 to 3 months after treatment^4,19^.

The prognosis of endodontically treated teeth depends not only on the treatment *per se*, but also on the placement of coronal restorations^28^. This treatment includes the decision of whether or not using intraradicular posts. Dentin loss may result in tooth fracture after the final restoration, and thus intracoronal strengthening is important in protecting the teeth against fracture^7,14^.

The industrial development of polymer-based composites and laminated structures has provided dental researchers with bondable, fractureresistant, esthetic, and extremely manageable reinforcement materials with ultra-high tensile strength that have the potential to be applied in dental restoration^21^. Since 1991, polyethylene fibers have been successfully used in a variety of clinical techniques^11,21^. The use of polyethylene fibers to restore endodontically treated teeth has gained popularity as an alternative to casts or prefabricated metal posts. This may be attributed to two important characteristics of fiber posts: their modulus of elasticity, which is similar to that of dentin, and their ability to be cemented^8,14^. It is believed that monoblock dentin-post-core systems with dentinal bonding enhance the distribution of forces along the root. Therefore, if excessive loads were applied to the tooth, the post would be able to absorb the stress, reducing the possibility of root fracture^8^. It is postulated that polyethylene fibers have a stress-modifying effect along the restoration-dentine interface. In addition, because fiber-reinforced posts are metal free, they do not cause metal allergies or corrode, and good esthetics in visible areas of the mouth. Finally, fiber-reinforced posts can be removed easily in the case of failure requiring retreatment^26^.

Endodontically treated anterior teeth with minimal loss of tooth structure may be restored conservatively with a bonded restoration in the access cavity^10,25,26^.However, root-filled teeth with exaggerated structural loss must treated using a complete crown for structural stability^3^. In a 3-year clinical study, Mannocci, et al.^3^ (2002) concluded that the clinical success rates of endodontically treated premolars with limited loss of tooth structure restored with fiber-reinforced posts and direct composite were equivalent to those restored with full-coverage metal-ceramic crowns.

Unless the majority of natural tooth substance remains after endodontic treatment, it is probably safer to provide some kind of cuspal coverage in the final coronal restoration, because most teeth that require endodontic treatment are usually damaged severely as a result of caries or fracture^26^.

In the patient presented in this paper, metalceramic restorations were preferred because the marginal ridges were lost due to extensive loss of tooth structure from caries and fracture and thus cuspal protection was necessary for long-term preservation of the remaining tooth structure. In conclusion, periapical lesions can respond favorably to nonsurgical endodontic treatment in young patients. Morever, with proper indication, polyethylene fibers can provide an effective conservative and esthetic option for reinforcing endodontically treated teeth undergoing prosthetic rehabilitation. These new fiber-reinforced posts may offer impressive results, but long-term clinical evaluation is necessary.
